# Supplementary figures and images for: Identification of suitable target/E3 ligase pairs for PROTAC development using a rapamycin-induced proximity assay (RiPA)
Source: eLife. 2024 Dec 6;13:RP98450. doi: 10.7554/eLife.98450 (PMC11623929; doi:10.7554/eLife.98450)

Figure 1 – Source data 1

D

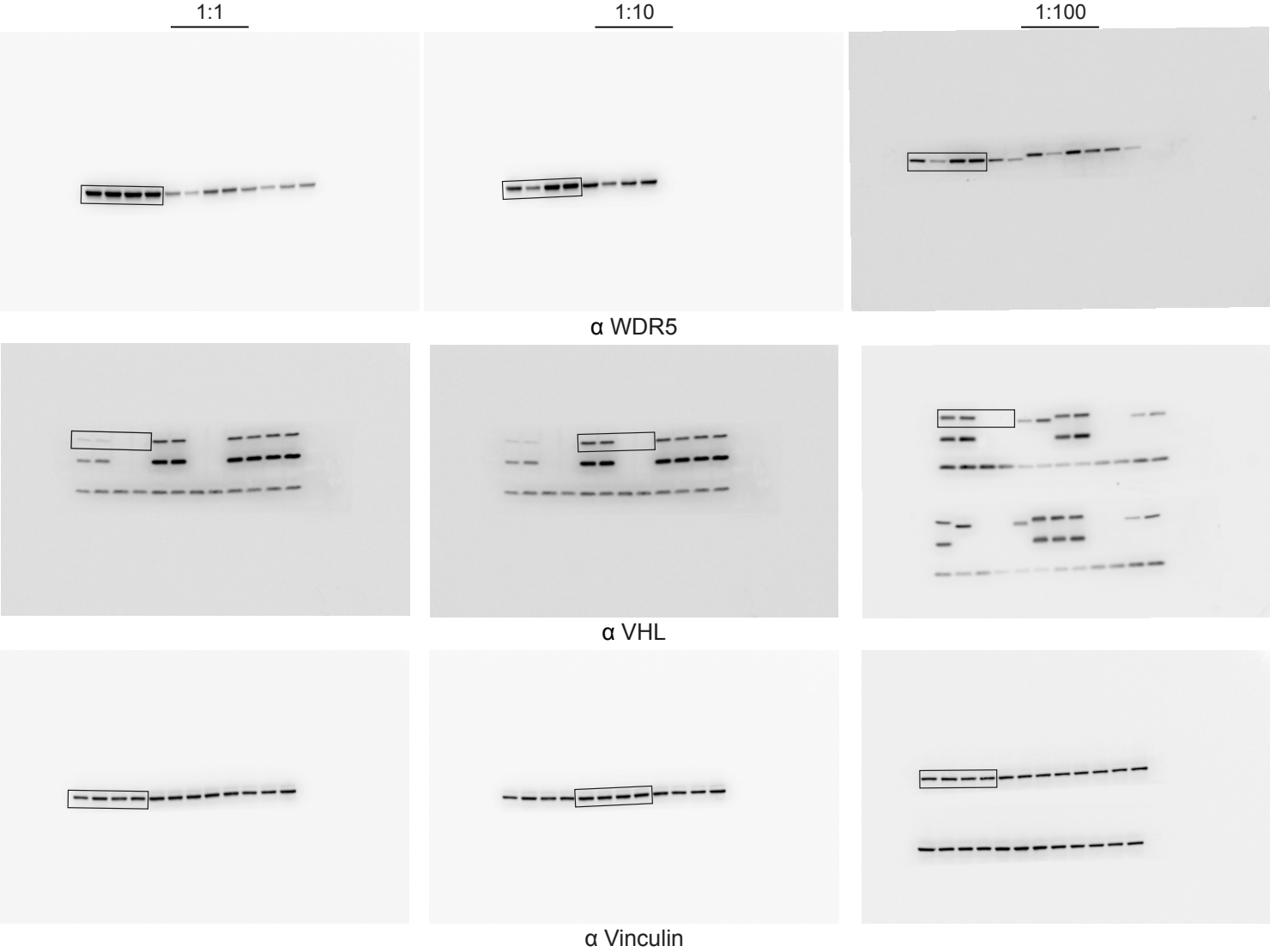

F

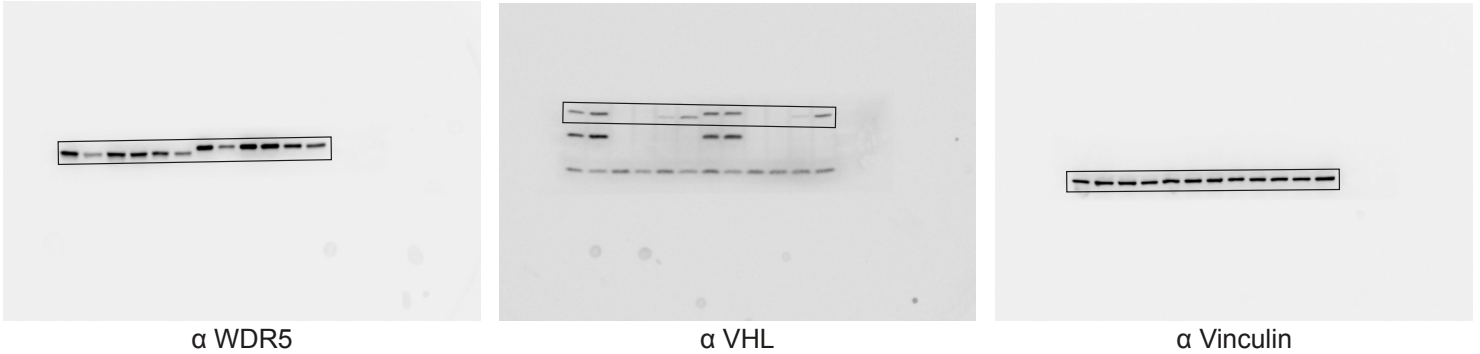

Supplement: Figure 1—source data 1. [file elife-98450-fig1-data1.zip › Figure 1ΓÇôsource data 1.pdf]

Figure 2 – Source data 1

A

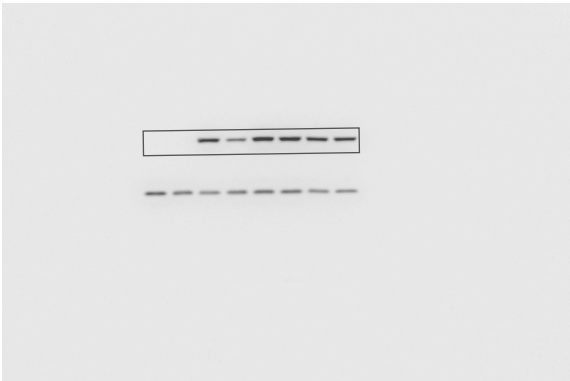

α WDR5

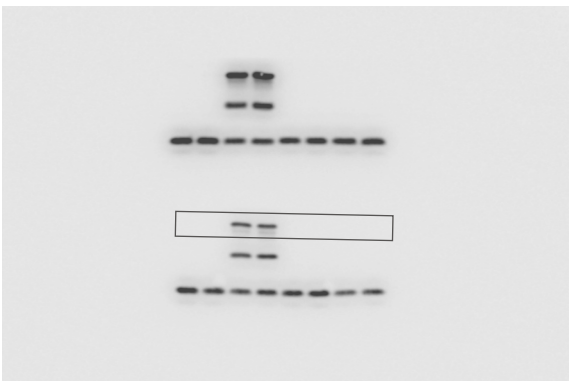

α VHL

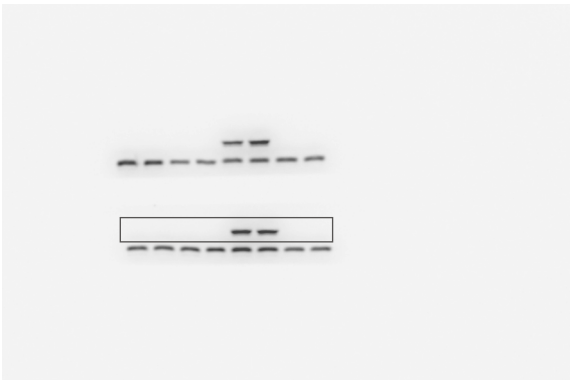

α CRBN

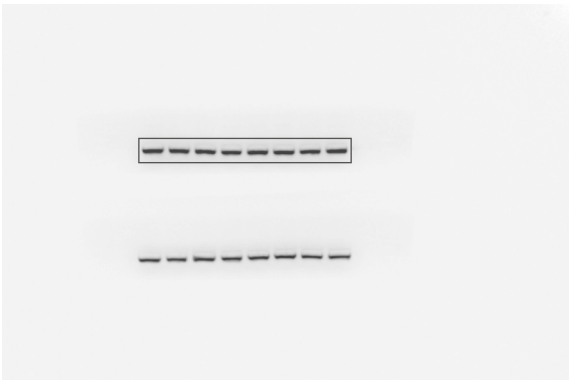

α Vinculin

C

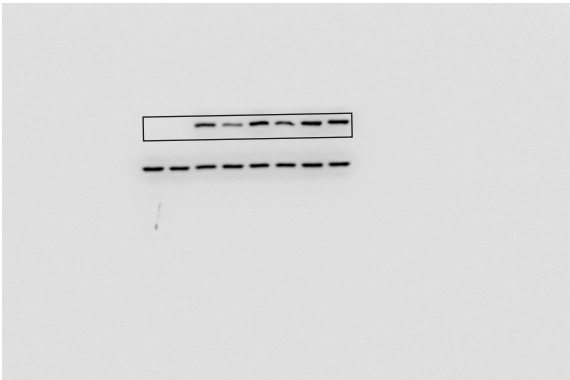

α AURKA

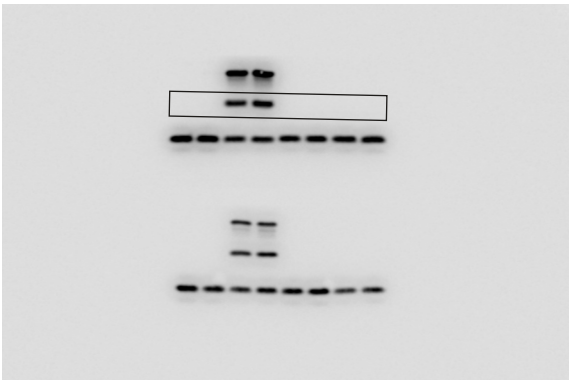

α VHL

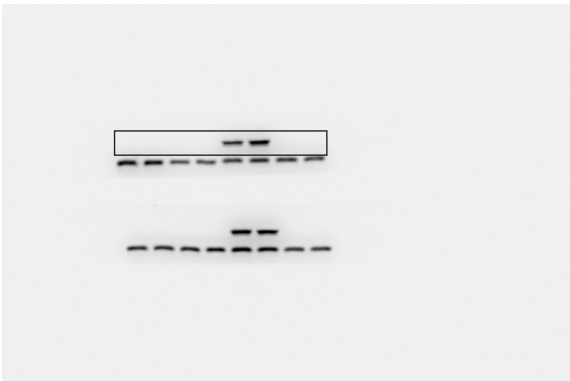

α CRBN

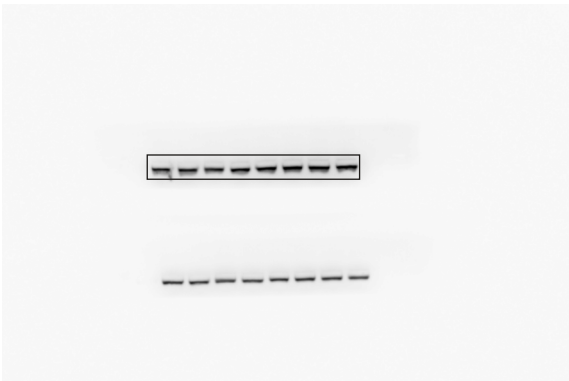

α Vinculin

Supplement: Figure 2—source data 1. [file elife-98450-fig2-data1.zip › Figure 2ΓÇôsource data 1.pdf]

Figure 2 - Figure supplement 1 – Source data 1

A

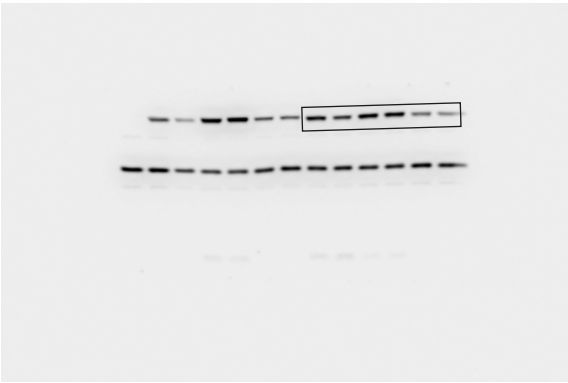

$\alpha$  WDR5

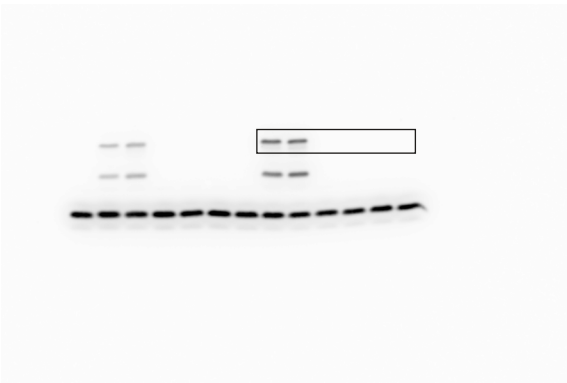

$\alpha$  VHL

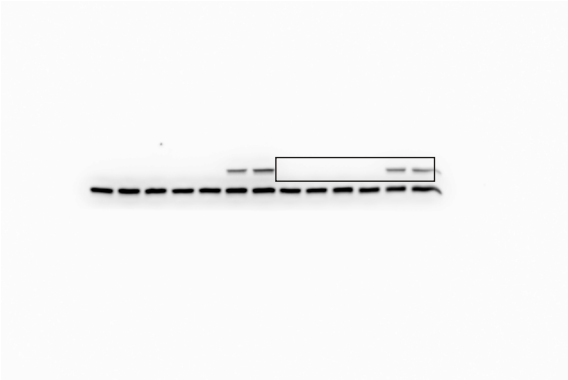

$\alpha$  CRBN

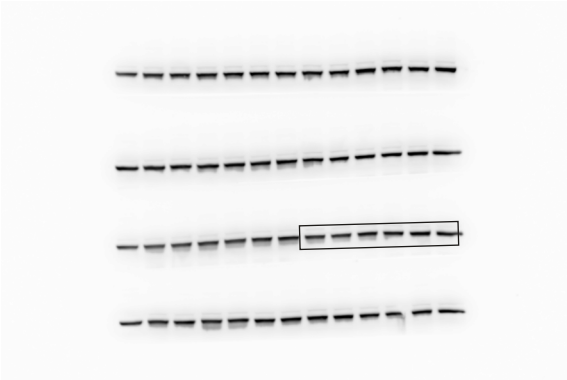

$\alpha$  Vinculin

Supplement: Figure 2—figure supplement 1—source data 1. [file elife-98450-fig2-figsupp1-data1.zip › Figure 2 - Figure supplement 1ΓÇôsource data 1.pdf]

Figure 3 – Source data 1

A

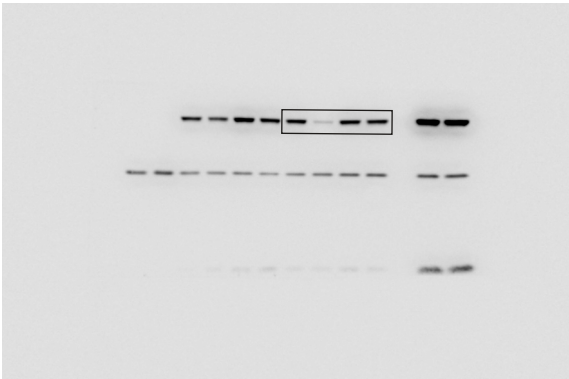

$\alpha$  WDR5

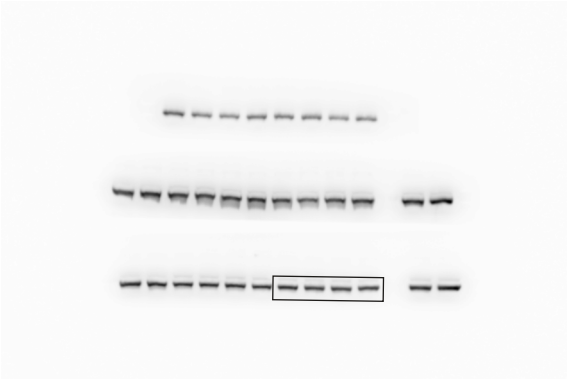

$\alpha$  Vinculin

E

FBXL12-FRB

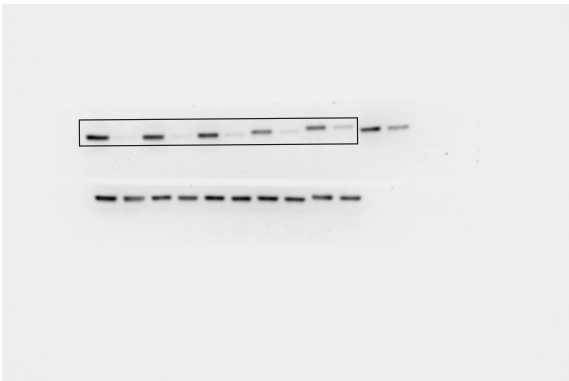

$\alpha$  WDR5

FRB

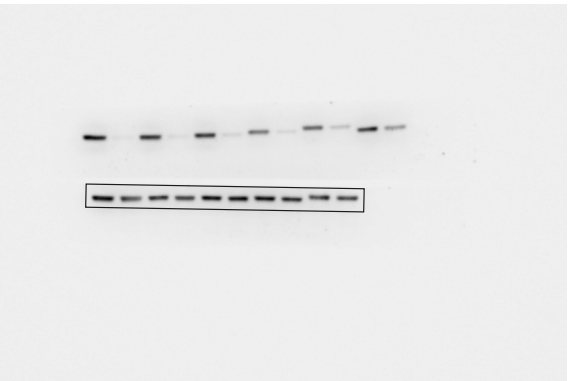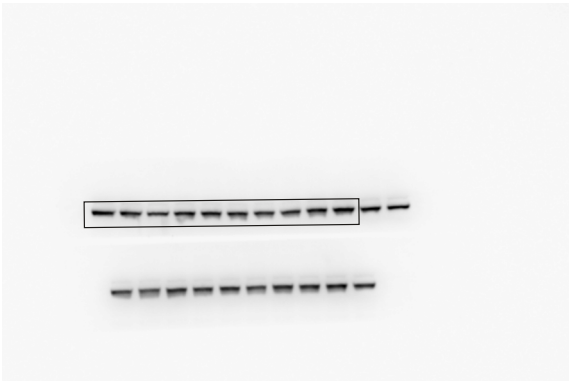

$\alpha$  Vinculin

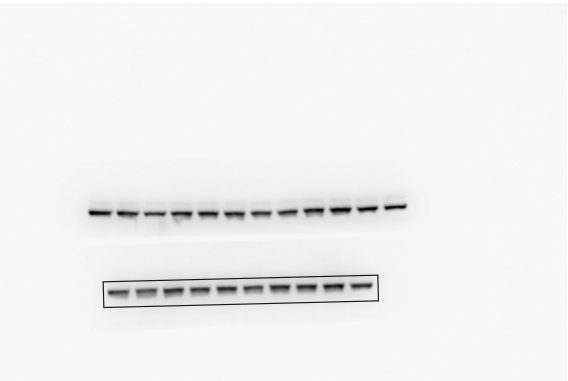

Supplement: Figure 3—source data 1. [file elife-98450-fig3-data1.zip › Figure 3ΓÇôsource data 1.pdf]
